# Identification of lipid droplets in gut bacteria

**DOI:** 10.1093/procel/pwac015

**Published:** 2022-07-15

**Authors:** Kai Zhang, Chang Zhou, Zemin Li, Xuehan Li, Ziyun Zhou, Linjia Cheng, Ahmed Hammad Mirza, Yumeng Shi, Bingbing Chen, Mengwei Zhang, Liujuan Cui, Congyan Zhang, Taotao Wei, Xuelin Zhang, Shuyan Zhang, Pingsheng Liu

**Affiliations:** National Laboratory of Biomacromolecules, CAS Center for Excellence in Biomacromolecules, Institute of Biophysics, Chinese Academy of Sciences, Beijing 100101, China; National Laboratory of Biomacromolecules, CAS Center for Excellence in Biomacromolecules, Institute of Biophysics, Chinese Academy of Sciences, Beijing 100101, China; School of Kinesiology and Health, Capital University of Physical Education and Sports, Beijing 100191, China; School of Kinesiology and Health, Capital University of Physical Education and Sports, Beijing 100191, China; National Laboratory of Biomacromolecules, CAS Center for Excellence in Biomacromolecules, Institute of Biophysics, Chinese Academy of Sciences, Beijing 100101, China; National Laboratory of Biomacromolecules, CAS Center for Excellence in Biomacromolecules, Institute of Biophysics, Chinese Academy of Sciences, Beijing 100101, China; National Laboratory of Biomacromolecules, CAS Center for Excellence in Biomacromolecules, Institute of Biophysics, Chinese Academy of Sciences, Beijing 100101, China; University of Chinese Academy of Sciences, Beijing 100049, China; Hebei Normal University, Shijiazhuang 050024, China; Hainan University, Haikou 570228, China; National Laboratory of Biomacromolecules, CAS Center for Excellence in Biomacromolecules, Institute of Biophysics, Chinese Academy of Sciences, Beijing 100101, China; National Laboratory of Biomacromolecules, CAS Center for Excellence in Biomacromolecules, Institute of Biophysics, Chinese Academy of Sciences, Beijing 100101, China; National Laboratory of Biomacromolecules, CAS Center for Excellence in Biomacromolecules, Institute of Biophysics, Chinese Academy of Sciences, Beijing 100101, China; University of Chinese Academy of Sciences, Beijing 100049, China; National Laboratory of Biomacromolecules, CAS Center for Excellence in Biomacromolecules, Institute of Biophysics, Chinese Academy of Sciences, Beijing 100101, China; University of Chinese Academy of Sciences, Beijing 100049, China; School of Kinesiology and Health, Capital University of Physical Education and Sports, Beijing 100191, China; National Laboratory of Biomacromolecules, CAS Center for Excellence in Biomacromolecules, Institute of Biophysics, Chinese Academy of Sciences, Beijing 100101, China; National Laboratory of Biomacromolecules, CAS Center for Excellence in Biomacromolecules, Institute of Biophysics, Chinese Academy of Sciences, Beijing 100101, China; University of Chinese Academy of Sciences, Beijing 100049, China

Dear Editor,

The field of gut microbiota is progressing rapidly and thus, appellations like the last human organ, a separate organ, a forgotten organ, and a new organ have been applied to gut microbiota to emphasize its vital functions in our body (Chang and Kao, 2019). Gut microbiota has been shown to play a central role in the regulation of human lipid metabolism and be associated with lipid metabolism disorders when aberrant, through composition and microbial metabolites. For instance, alterations in intestinal microbiota composition are associated with dyslipidemia and ectopic fat deposition in host. Moreover, transplantation of the fecal microbiota from donors with obesity, type 2 diabetes, or steatosis would partially result in similar metabolic phenotype in mouse recipients (Schoeler and Caesar, 2019). The metabolites produced by gut microbiota, such as short-chain fatty acids which provide energy for intestine and contribute to lipogenesis in liver, may mediate the influence on host lipid metabolism (Schoeler and Caesar, 2019).

Several sophisticated approaches have been established to study gut microbiota, including high-throughput 16S rRNA sequencing, metagenomics, metabolomics, and massive bacterial cloning. The immense number of species in the gut and the resulting complexity have stymied efforts to elucidate the functions of the microbiota (Lozupone et al., 2012). In comparison, other human organs and tissues are composed of well-studied cell types containing known organelles with a developed understanding of their molecular regulation and communication. However, these foundations have not yet been built for gut microbiota. Therefore, new techniques and approaches are required to dissect this important ecosystem and elucidate their cellular communication, cellular organelles, and molecular regulation.

The lipid droplet (LD) is a cellular organelle with a globular shape and consists of a neutral lipid core covered by a monolayer phospholipid membrane decorated with resident/dynamic proteins (Olzmann and Carvalho, 2019). Abnormal LD dynamics and excess lipid storage in mammalian LDs have been directly linked to many metabolic syndromes, including obesity, fatty liver, atherosclerosis, and diabetes (Xu et al., 2018). Besides mammalian cells, many bacteria contain LDs, with lipophilic compounds, such as polyhydroxybutyrate (PHB), triacylglycerol (TAG), and wax ester (WE), in the core (Murphy, 2012). Several proteins on bacterial LDs have been identified, such as microorganism lipid droplet small (MLDS) and phage shock protein A (PspA) (Ding et al., 2012; Chen et al., 2014). Bacterial LDs have been found to be vital in multiple processes. The accumulation of PHB, TAG, and WE in bacterial LDs is provoked in response to stress, such as high carbon and low nitrogen availability (Waltermann and Steinbuchel, 2005). Besides, LDs in pathogenic bacteria play an important role in the process of pathogenesis. For instance, accumulation of LDs in *Mycobacterium tuberculosis* would provide fuels for host colonization (Daniel et al., 2011). Bacterial LDs would bind DNA via MLDS to enhance survival (Zhang et al., 2017).

The gut microbiota has been proven to perform various key functions in the human body and it is known to be dominated by bacteria (Derrien et al., 2019). Therefore, it is of interest to investigate how gut bacteria subsist and what molecules they may produce that affect human health. As a first step we decided to determine whether some gut bacteria have LDs.

Although LDs have been identified in several cultured bacterial strains *in vitro*, they have never been visualized in bacteria *in situ*. As it is technically possible to examine gut bacteria within the mouse intestine we first analyzed gut bacteria *in situ* in mice fed a regular chow to determine if LDs could be visualized. Ultrastructure was examined by transmission electron microscopy (TEM) revealing low-density spherical structures indicated by the red arrows, demonstrating for the first time the presence of LDs in bacteria in mouse small intestine, large intestine, and feces (Fig. 1A).

**Figure 1.**
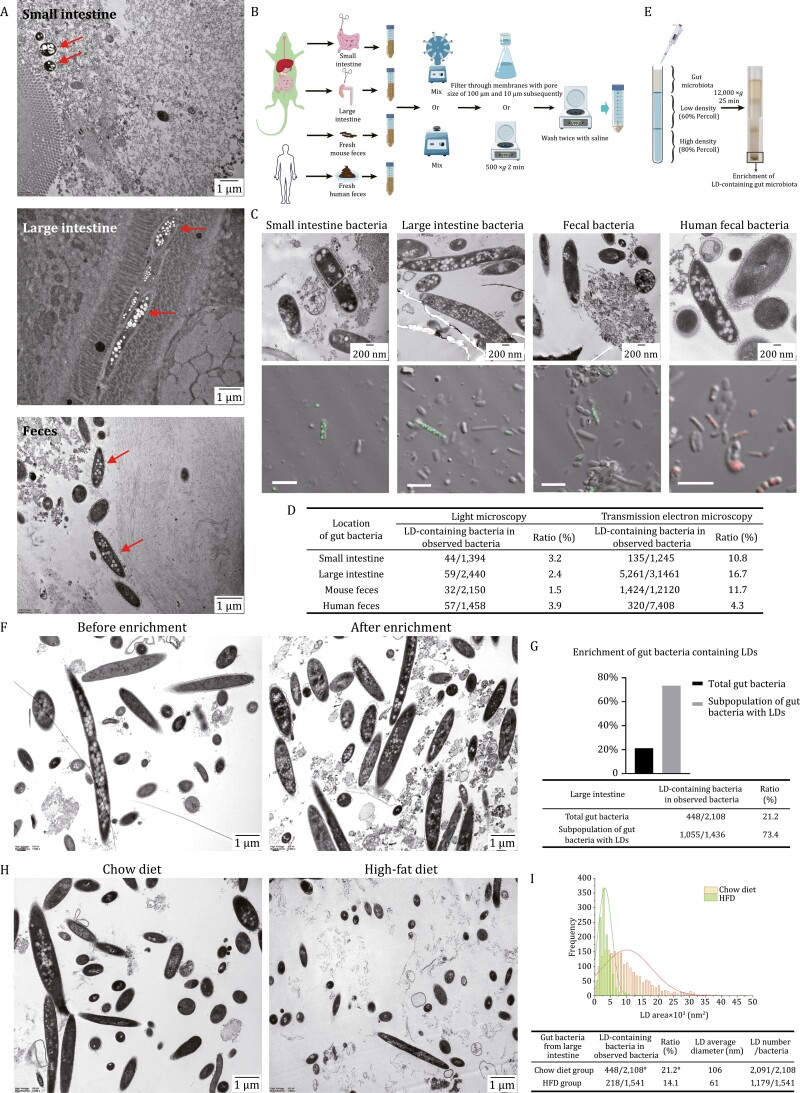
LDs in gut bacteria. (A) Ultrastructural analysis of mouse intestinal and fecal bacteria *in situ* by TEM. Briefly, the freshly collected intestine or feces were fixed immediately and embedded in agarose. Then the samples were postfixed in 2% potassium manganate followed by ethanol dehydration and resin embedding. 70 nm ultra-thin sections were transferred onto grids and were stained with uranyl acetate and lead citrate for visualization under TEM. Top, mouse small intestine; middle, mouse large intestine; bottom, mouse feces. Scale bar, 1 µm. (B) The workflow for isolation of microbiota from mouse small intestine, large intestine, mouse and human feces. In brief, mouse small and large intestines and their contents, and the mouse and human fresh feces were collected. Samples were transferred into 50 mL centrifuge tubes and fully mixed in saline by rotation or vortexing. Finally, the samples were filtered or centrifuged to remove food residue, and the bacteria were concentrated and washed two times for use. The illustration used some elements from Servier Medical Art. (C and D) LDs in isolated gut bacteria analyzed by TEM and confocal microscope. (C) Briefly, gut bacteria were isolated freshly from mouse small and large intestines, mouse and human feces, and subjected immediately to sample preparation for TEM and neutral lipid staining. Ultrastructures of the freshly isolated bacteria were presented in upper panel. Briefly, the freshly isolated bacteria were fixed immediately and prepared as ultra-thin sections (70 nm) following dehydration in a series of ethanol, embedding into epoxy resin and sectioning. After staining, the sections were observed under TEM. Scale bar, 200 nm. Neutral lipid staining of fresh gut bacteria were presented in lower panel. Briefly, the isolated bacteria were stained with LipidTOX Green or Red, and imaged by confocal microscope. Scale bar, 5 µm. Then the ratio of LD-containing gut bacteria was calculated as the number of LD-containing bacteria to the number of bacteria counted. (D) 20–50 confocal images for each sample were taken to count the bacteria. All the bacteria in the ultra-sections for each sample were counted in TEM study. (E) The workflow for enrichment of LD-containing gut bacteria. Briefly, after isolation of gut bacteria from mouse large intestine, the bacteria were separated further through Percoll density gradient centrifugation to enrich the subpopulation enriched in LDs. (F and G) Enrichment of LD-containing gut bacteria revealed by TEM. (F) Gut bacteria from mouse large intestine (before enrichment, left panel) and the subpopulation enriched in LDs (after enrichment, right panel), obtained through Percoll density gradient centrifugation, were viewed through TEM. Scale bar, 1 µm. Around 60 TEM images for each group were taken to calculate the ratio of bacteria containing LDs (G). (H and I) LDs in gut bacteria from mice fed chow diet or high-fat diet. Eight-week-old mice given *ad libitum* access to water and chow diet were randomly divided into two groups of three each. One group was fed chow diet and another high-fat diet (HFD, 60% energy from fat). After 4 weeks, the mice were sacrificed and gut bacteria of large intestine were isolated and viewed by TEM. (H) The representative images of isolated bacteria from mice fed chow diet (left panel) and HFD (right panel) are presented. Scale bar, 1 µm. Around 60 TEM images for each group were taken to calculate the ratio of bacteria containing LDs, and analyze the LD number, size, and area. (I) The LD area, number, and size in gut bacteria of each group were estimated using Imaris v9.8, and a diameter threshold (>15 nm) for LDs was set. ^#^ means the data are from (G).

To confirm this finding, we isolated bacteria from mouse small and large intestines, as well as from mouse and human feces using a method described in Fig. 1B. To visualize the morphology of LDs, the freshly isolated bacteria were fixed immediately and subjected to ultrastructural analysis using TEM. Low-density spherical structures were detected inside of some bacteria isolated from small intestine (Fig. 1C, upper panel, Fig. S1A), large intestine (Fig. 1C, upper panel, Fig. S2A), mouse feces (Fig. 1C, upper panel, Fig. S3A), and human feces (Fig. 1C, upper panel, Fig. S4A).

To determine if these structures contained neutral lipids, a key characteristic of LDs, samples of the isolated bacteria were stained with a neutral lipid dye LipidTOX Red/Green and imaged by confocal microscopy with differential interference contrast images to identify bacteria. Overlapping images represent neutral lipids inside of bacteria (Ding et al., 2012). Examination of the isolations demonstrated that some bacteria showed positive fluorescent signals, including bacteria isolated from mouse small intestine (Fig. 1C, lower panel, Fig. S1B), mouse large intestine (Fig. 1C, lower panel, Fig. S2B), mouse feces (Fig. 1C, lower panel, Fig. S3B), and human feces (Fig. 1C, lower panel, Fig. S4B). This suggests that those structures in gut bacterial cells contained neutral lipids. The fluorescent signals and TEM pictures revealed that LDs were in a variety of sizes and numbers within bacteria with a range of morphologies, suggesting the diversity of not only gut bacteria but also their LDs.

Further, based on our collections, we counted 20–50 confocal images and almost all the gut bacteria viewed under TEM to calculate the ratio of bacteria containing LDs and the results are summarized in Fig. 1D. As shown in the TEM images (Figs. 1A, 1C and S1–S4), many LDs in gut bacteria were smaller than 100 nm which is the resolution limit of light microscopy. Consistently, more LDs were viewed under TEM than confocal microscopy (Fig. 1C). These data show that how to accurately quantify the size and number of LDs is critical when studying LDs in gut bacteria.

More importantly, those bacteria containing LDs could be enriched through Percoll density gradient centrifugation (Fig. 1E). Briefly, after isolation of gut bacteria from mouse large intestine (Fig. 1F, left panel, Fig. S5A), the gut bacteria were loaded to the top of a Percoll density gradient and centrifuged. TEM analysis revealed that around 75% of the subpopulation at the bottom of the centrifuge tube were shown to contain LDs (Fig. 1F, right panel, G, Fig. S5B). The enrichment of gut bacteria containing LDs further verified the existence of LDs in gut bacteria, and moreover, provided a crucial way to illustrate the property and function of those bacteria containing LDs.

Since LDs are essential in cellular metabolism and LDs exist in gut bacteria, we wondered whether the gut bacterial LDs would be affected by host diet. We fed mice on a normal chow or high-fat diet for 4 weeks. Similar to the previous reports, gut bacteria were reduced significantly in amount when mice were fed on high-fat diet (Fig. 1H and S6; Singh et al., 2017). More importantly, TEM analysis showed that the ratio of gut bacteria containing LDs in mice fed on high-fat diet decreased and LDs were smaller and less (Fig. 1H and 1I), suggesting a possible role those LDs may play in regulation of host metabolism.

All above data present LDs exist and neutral lipid are contained in gut bacteria. A comprehensive method for studying a cellular organelle, of course, should include imaging, purification, and omics approaches. Therefore, two cloned strains of human gut bacteria were collected, cultivated, and analyzed. One culture was *Rhodococcus erythropolis*, a species previously identified in human colon (Lepage et al., 2011), and *Streptomyces thermovulgaris*, which was previously isolated from human fecal samples (Dubourg et al., 2013). Both bacteria were found to have LDs using TEM (Fig. 2A and 2G) and to have neutral lipid-positive structures by confocal microcopy (Fig. 2B and 2H).

**Figure 2.**
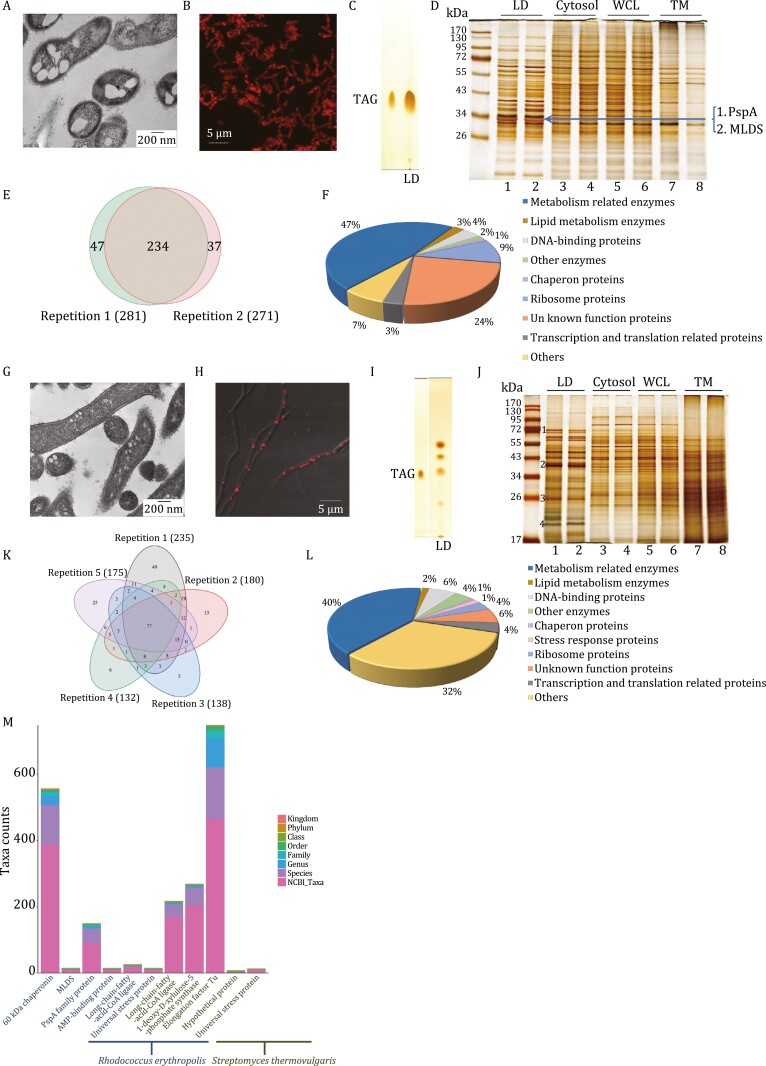
Identification and analysis of LDs in cloned gut bacteria. (A–D) A strain of human gut bacteria, *Rhodococcus erythropolis*, was used for analysis of LD in gut bacteria. *R. erythropolis* was cultured in MSM medium. (A) Ultra-thin structural analysis of *R. erythropolis* by TEM. Briefly, the bacteria were fixed immediately after collection, dehydrated, and embedded, and finally sectioned into ultra-thin sections (70 nm). The sections were stained and viewed by TEM. Scale bar, 200 nm. (B) Confocal images of *R. erythropolis* were captured after neutral lipid staining with LipidTOX Red. Scale bar, 5 µm. LDs were then isolated from *R. erythropolis* using the method we previously reported (Ding et al., 2012). (C) Neutral lipids of isolated LDs were analyzed by thin layer chromatography (TLC). Lane 1, TAG marker; Lane 2, LDs of *R. erythropolis*. Then the proteins of the LDs isolated from *R. erythropolis* were studied through biochemical methods and proteomic analysis. (D) The protein profiles of the cellular fractions of *R. erythropolis* were analyzed by SDS–PAGE and silver staining. The major band in the gel indicated was sliced and subjected to in-gel digestion followed by LC/MS/MS identification. TM, total membrane; WCL, whole cell lysate. (E and F) Two LD isolations from two independent cultures of *R. erythropolis* were conducted. The total LD proteins of each isolation were subjected to proteomic identification. (E) A Venn diagram was built to present the overlap of the identified LD proteins of the two biological replicates. (F) The identified LD proteins from LC/MS/MS were then analyzed using bioinformatics tools and the proteins with two unique peptides or above were selected. Based on their functions, they were categorized into nine groups. (G–J) The other strain of human gut bacteria, *Streptomyces thermovulgaris*, was used for analysis of LD in gut bacteria. *S. thermovulgaris* was cultured in ISP2 medium. (G) Ultra-thin structural analysis of *S. thermovulgaris* by TEM. Briefly, the bacteria were fixed immediately after collection, dehydrated, embedded, and finally sectioned into ultra-thin sections (70 nm). The sections were stained and viewed by TEM. Scale bar, 200 nm. (H) Confocal images of *S. thermovulgaris* were captured after neutral lipid staining with LipidTOX Red. Scale bar, 5 µm. LDs were isolated. (I) Neutral lipids of isolated LDs were analyzed by TLC. Lane 1, TAG marker; Lane 2, LDs of *S. thermovulgaris*. The proteins of the isolated LDs from *S. thermovulgaris* were studied using biochemical methods and proteomic analysis. (J) The protein profiles of the cellular fractions of *S. thermovulgaris* were analyzed by SDS–PAGE and silver staining. The four major bands indicated were sliced and subjected to in-gel digestion followed by LC/MS/MS identification. TM, total membrane; WCL, whole cell lysate. (K and L) Five LD isolations from five independent cultures of *S. thermovulgaris* were conducted. The total LD proteins of each isolation were subjected to proteomic identification independently. (K) A Venn diagram was built to present the overlap of the identified LD proteins of the five biological replicates. (L) The identified LD proteins from LC/MS/MS were then analyzed using bioinformatics tools and the proteins with two unique peptides or above were selected. Based on their functions, they were categorized into 10 groups. (M) Taxonomic dissection and informatics analysis of several LD abundant proteins of gut bacteria. The taxonomic composition distribution histograms at each classification level of the LD abundant proteins from gut bacteria *R. erythropolis* and *S. thermovulgaris* are presented. The Kraken2 database was used and the sequences with identity values above or equal to 80% were screened.

The LDs were isolated by flotation on a density gradient using our established method (Ding et al., 2012). The LD fraction was washed to remove possible contamination and analyzed to determine the purification quality. The lipids were extracted and analyzed using thin layer chromatography and TAG was identified in LDs from both bacteria (Fig. 2C, lane LD and Fig. 2I, lane LD). Interestingly, two unknown neutral lipids were also identified in the LDs of *S. thermovulgaris* (Fig. 2I, lane LD). For both bacteria, the protein composition of the LDs was distinct from cytosol (Cytosol), whole cell lysate, and total membrane (Fig. 2D and 2J). Analysis of the protein composition from two independent isolations illustrates the reproducibility of the purification (Fig. 2D, Lanes 1 and 2, and Fig. 2J, Lanes 1 and 2). Collectively, the results demonstrate that the purification method produces high-quality preparations of LDs. Next the proteins of the LDs isolated from the two cultures were studied in detail through proteomics.

Analysis of the *R. erythropolis* LDs was first conducted by in-gel digestion of the most prominent band with analysis by LC/MS/MS. The two most abundant proteins were PspA and MLDS (Fig. 2D and Table S1), both previously reported in bacterial LDs (Ding et al., 2012; Chen et al., 2014). Total sample proteomics of LD proteins was also conducted identifying 318 proteins (Table S2) including 234 common to duplicate preparations (Fig. 2E and Table S3). All 318 identified proteins were categorized into 9 groups (Fig. 2F and Table S4).

There were four major bands in the *S. thermovulgaris* LDs analyzed by in-gel digestion (Fig. 2J). The proteins identified are listed in Table S5. Total protein analysis was also conducted on five independent preparations (Table S6). There were 77 proteins common to all analyses (Fig. 2K and Table S7). The total number of proteins identified was 310 and these proteins were categorized into 10 groups according to their functions (Fig. 2L and Table S8).

These data confirm the presence of LDs in two human gut bacterial species, *R. erythropolis* and *S. thermovulgaris*. Collectively, the data presented demonstrate the existence of LDs in gut bacteria.

To further investigate how common LDs are in gut bacterial species we analyzed the taxonomic distribution of the most abundant LD proteins identified in the two cloned bacteria. First, the sequence number of the proteins at each classification was calculated (Fig. 2M). The result showed that 60 kDa chaperonin and elongation factor-Tu (EF-Tu) were widely expressed in bacteria. PspA, 1-deoxy-d-xylulose-5-phosphate synthase, and ­long-chain-fatty-acid-CoA ligase were also relatively common. Other proteins were not common in bacteria. PspA and EF-Tu were further dissected at the genus level (Fig. S7), suggesting that these bacteria have LDs and LDs are common in gut bacteria.

Six abundant proteins/genes were subjected to functional informatics analysis using their eukaryotic homologs (listed in Table S9). Gene concurrence analysis showed distribution of these six genes across Bacteria, Archaea, and Eukarya, the three domains of life (Fig. S8Aa). Further analysis was performed examining gene coexpression profiles (Fig. S8Ab). In addition, Fig. S8B presents that total five clusters were made between the six selected proteins.

In summary, we demonstrated the existence of LDs in mouse and human gut bacteria and the gut bacterial LDs. Our data also indicate that LDs in gut bacteria may be involved in host health. Further we elucidated the LD proteins and lipids of two cloned human gut bacteria, and LDs are predicted to be common in gut bacteria. Moreover, the gut bacteria containing LDs could be enriched. These new findings allow researchers to investigate gut bacteria from a completely new angle and provide a new approach at the organelle level to study gut bacteria. Based on current understanding of the organelle, it can be expected that LD in gut bacteria plays important roles in gut functions including food digestion and absorption, production of essential molecules, modification of the gut environment, immune protection, detoxification, and fecal energy output, which are fundamental to human health.

## Supplementary Material

pwac015_suppl_Supplementary_Material

pwac015_suppl_Supplementary_Tables

## Footnotes

The authors thank Dr. John Zehmer for his critical reading and useful suggestions. The authors thank Ms. Xixia Li and Ms. Xueke Tan for helping with electron microscopy sample preparation, Ms. Yan Teng for her help of taking confocal images, and Ms. Yun Feng for their help of analyzing TEM images, at the Center for Biological Imaging (CBI), Institute of Biophysics, Chinese Academy of Science. The authors thank Ms. Zhensheng Xie for her technical supports of proteomic study.This work was supported by the National Natural Science Foundation of China (Grant Nos. 91857201 and 91954108), and the Ministry of Science and Technology of the People's Republic of China (Grant Nos. 2018YFA0800700 and 2018YFA0800900).

The original data that support the findings of this study are available from the corresponding author upon request.

The authors declare that they have no competing interests.

All institutional and national guidelines for the care and use of laboratory animals were followed.

Informed consent was obtained from all individual participants included in the study.

P.L., S.Z. and X.Z. designed the project. K.Z., S.Z., C.Z., L.C., Z.L., and X.L. performed the experiments. Z.Z. and A.H.M. carried out bioinformatics analysis. Y.S. and B.C. helped with analysis of TEM images. Z.L. and M.Z. helped with triacylglycerol assay. X.Z., C.Z., L.C. and T.W. helped with MS study. P.L., S.Z., and C.Z. wrote the manuscript. All authors have read and approved the manuscript.
