# Supplementary material for: Identification of lipid droplets in gut bacteria
Source: Protein Cell. 2022 Jul 15;14(2):143–8. doi: 10.1093/procel/pwac015 (PMC10019568; doi:10.1093/procel/pwac015)
Supplement: pwac015_suppl_Supplementary_Material [file pwac015_suppl_supplementary_material.docx]

**Supplemental information**

**Identification of Lipid Droplets in Gut Bacteria**

Kai Zhang^1✢^, Chang Zhou^1✢^, Zemin Li^2✢^, Xuehan Li^2✢^, Ziyun Zhou^1^, Linjia Cheng^1^, Ahmed Hammad Mirza^1,3^, Yumeng Shi^4^, Bingbing Chen^5^, Mengwei Zhang^1^, Liujuan Cui^1^, Congyan Zhang^1,3^, Taotao Wei^1,3^, Xuelin Zhang^2^*, Shuyan Zhang^1^*, and Pingsheng Liu^1,3^*

^1^National Laboratory of Biomacromolecules, CAS Center for Excellence in Biomacromolecules, Institute of Biophysics, Chinese Academy of Sciences, Beijing 100101, China

^2^School of Kinesiology and Health, Capital University of Physical Education and Sports, Beijing 100191, China

^3^University of Chinese Academy of Sciences, Beijing 100049, China

^4^Hebei Normal University, Shijiazhuang 050024, China

^5^Hainan University, Haikou 570228, China

^✢^These authors contributed equally.

*To whom correspondence should be addressed.

Address: 15 Datun Road, Beijing 100101, China

E-mail: pliu@ibp.ac.cn, zhangxuelin@cupes.edu.cn, and syzhang@ibp.ac.cn

**Materials and Methods**

**Materials and bacterial strains**

The LipidTOX Red/Green dyes were from Invitrogen. Lipid standards were from Sigma. 25% glutaraldehyde solution (EM grade), 8% paraformaldehyde solution (EM grade), Embed 812 kit, uranyl acetate and lead citrate were all purchased from Electron Microscopy Sciences (Hatfield, USA). Osmium tetraoxide (EM grade) was purchased from Nakalai Tesque (Kyoto, Japan). Potassium permanganate was purchased from Merck (Darmstadt, Germany). The *Streptomyces thermovulgaris* and *Rhodococcus erythropolis* strains were purchased from China General Microbiological Culture Collection Center. Percoll was purchased from GE Healthcare Bio-Sciences AB (Uppsala. Sweden).

**Isolation bacteria freshly from mouse gut and feces**

All animal experiments were approved by the Committee of Biosafety, Ethics and Experimental Animal Management of Institute of Biophysics, Chinese Academy of Sciences, permit number SYXK (Jing) 2016-0026, and were performed in accordance with the NIH Guide for the Care and Use of Laboratory Animals (8th edition). Eight-week-old C57BL/6 mice were purchased from Beijing Vital River Laboratories. The mice were given ad libitum access to chow diet and water.

The gut bacteria were isolated according to the method previously reported with modification^1^. Briefly, the fresh feces (1 fecal sample: minimum 0.2 g) were collected 10 min before decapitation. After decapitation, small intestine and large intestine were resected and the mesentery was removed with careful dissection. The small intestine and large intestine were carefully slit open with surgical scissors. The contents of small intestine and large intestine, as well as mouse feces were transferred immediately into saline (4°C, 20 mL) in 50 mL centrifuge tubes and rotated for 10 min at 4°C or vortexed for 5 min. The mixture was filtered through a suction filtration system with a 100-μm membrane and subsequently through a 10-μm membrane filter or centrifuged at 500*g* for 2 min at 4°C to remove food residue. The resulting sample solution was centrifuged at 3,200*g* for 20 min at 4°C. The pellet was resuspended with 1 mL cold saline and transferred into a 1.5 mL centrifuge tube and centrifuged at 10,000*g* for 2 min at 4°C. The washing step was repeated once more. The resulting bacteria were used for further analysis.

**Isolation of bacteria from fresh human feces**

Bacteria of fresh human feces were isolated in a manner similarly to the method described above. A fresh fecal sample (about 10 mL) was collected from a healthy 26-year-old man. The volunteer had no previous history of gastrointestinal disease and had not consumed antibiotics in the three months prior to this study. The fresh feces were transferred immediately into a 50 mL centrifuge tube and were resuspended in 30 mL cold saline. The sample was vigorously vortexed 5 times for 1 min, with 1-min intervals in between vortexing steps. The mixture was passed through a suction filtration system with a 100-μm membrane and subsequently a 10-μm membrane filter or centrifuged at 500*g* for 2 min at 4°C to remove food residue. The bacteria were collected and washed similar to the procedure described above for the mouse samples.

**Enrichment of gut bacteria containing lipid droplets**

After collection of fresh gut bacteria from mouse large intestine, the bacteria were further subdivided to enrich those containing LDs, through discontinuous Percoll density gradient centrifugation. In detail, 8 mL of 100% Percoll and 2 mL of cold saline were mixed to prepare 80% Percoll solution. Similarly, 60% Percoll solution was prepared by mixing 6 mL of 100% Percoll with 4 mL of cold saline. 3.5 mL of the 80% Percoll solution was first added to a round bottom polypropylene centrifuge tube (Beckman SW41 centrifuge tube, 14 × 89 mm). Then 3.5 mL of the 60% Percoll solution was layered gently. This discontinuous density gradient was kept on ice until use. The sharp interface between the two layers should be clearly discernable. Bacteria from large intestines of three mice were isolated as described above, and were resuspended with 5 mL cold saline. 3 mL of the resulting suspension was carefully added to the top of the density gradient and the tube was centrifuged at 12,000*g* for 25 min at 4°C. After centrifugation, three distinct bands and a pellet were produced. The upper solution was gently disposed and the pellet was carefully collected to a 1.5 mL centrifuge tube. The pellet was washed twice using saline by centrifugation at 12,000*g* for 2 min at 4°C. The resulting subpopulation of gut bacteria were viewed under TEM and most of them were found to contain lipid droplets.

**LipidTOX staining of isolated bacteria**

Freshly isolated bacteria were applied to poly-L-lysine (PB0589)-pretreated cover glasses before washing. The bacteria were then stained with LipidTOX Red/Green (1:500, v/v) for 30 min. The cover glasses were then mounted on glass slides using mounting media (P0126) and observed with a ZEISS LSM 980 confocal laser scanning microscope.

**Ultrastructural analysis of intestine and bacteria by transmission electron microscopy (TEM) *in situ* and *in vitro***

Ultrastructures of mouse small intestine, large intestine and feces were analyzed by TEM. The freshly excised intestines or collected feces were fixed immediately in glutaraldehyde (2.5%, v/v) and paraformaldehyde (2%, v/v) in 0.1 M PB (pH 7.2) and then embedded in 4% agarose. Then the samples were further fixed overnight at 4ºC. Subsequently they were post-fixed in 2% potassium permanganate for 1 h at room temperature. Then the samples were dehydrated in an ascending concentration series of ethanol followed by propylene oxide at room temperature. After dehydration, the samples were embedded in Embed 812 and prepared as 70-nm-thick ultrathin sections using Leica EM UC6 Ultramicrotome. Ultrathin sections were collected on formvar-coated copper grids and stained with uranyl acetate and lead citrate. The sections were then observed with a Tecnai Spirit electron microscope (FEI, Netherlands).

Ultrastructures of bacteria, both freshly isolated and cultured bacteria, were also analyzed using TEM after ultra-thin sectioning. Briefly, bacteria were pelleted and washed twice with the indicated buffer. For freshly isolated bacteria from gut and feces, 0.1 M PB (pH 7.2) was used and for *in vitro* cultured bacteria, 50 mM K-Pi (pH 7.2) was used. Then the cells were fixed in buffered glutaraldehyde (2.5%, v/v) and paraformaldehyde (2%, v/v) overnight at 4ºC. Subsequently, the cells were further fixed in 2% (w/v) potassium permanganate for 5 min at room temperature. Then the samples were dehydrated, embedded and finally prepared as 70-nm-thick ultrathin sections. The sections were stained and then observed with a Tecnai Spirit electron microscope (FEI, Netherlands).

**Analysis of LD size and number in gut bacteria**

Pictures of gut bacteria imaged under TEM were processed using Imaris (version 9.8, Bitplane) to analyze the size and number of LDs in gut bacteria. Briefly, Spots model in Imaris (red spheres) was used to estimate the number of lipid droplets per cell. Surfaces model in Imaris was used to estimate the size of lipid droplets.

**Cultivation of *Rhodococcus erythropolis* and *Streptomyces thermovulgaris***

*Rhodococcus erythropolis* was cultured in 10 mL MSM medium (0.5 g/L NH_4_Cl, 0.2 g/L MgSO_4_•7H_2_O, 0.02 g/L CaCl_2_•2H_2_O, 1.5 g/L KH_2_PO_4_, 0.0012 g/L C_6_H_8_FeNO_7_, 0.1 mL/L SL6, 9 g/L Na_2_HPO_4_•12H_2_O, 10 g/L D-glucose, pH 7.2) at 30°C. The growth of *Rhodococcus erythropolis* was monitored by OD_600_.

*Streptomyces thermovulgaris* was cultured in 10 mL ISP2 medium (1% Malt extract, 0.4% Yeast extract, 0.4% D-glucose, pH 7.2) at 37°C. The growth of *Streptomyces thermovulgaris* was monitored by determination of its dry weight.

**Lipid droplet isolation**

Lipid droplets of *Rhodococcus erythropolis* and *Streptomyces thermovulgaris* were purified according to our previously established method with modification^2^. In brief, bacteria were collected and washed twice with Buffer A (25 mM tricine, 250 mM sucrose, pH 7.8). After incubation in Buffer A for 20 min on ice, the bacteria were homogenized by passing through a French pressure cell six times at 1,200 bar, 4°C. The sample was then centrifuged at 6,000*g* for 10 min to remove cell debris and unbroken cells. The resulting supernatant (10 mL) was overlaid with 2 mL Buffer B (20 mM HEPES, 100 mM KCl, 2 mM MgCl_2_, pH 7.4) and centrifuged at 182,000*g* for 1.5 h at 4°C (Beckman SW40). Then the lipid droplets on the top were collected and washed for further analysis.

**Analysis of neutral lipids by thin layer chromatography (TLC)**

After collection, LDs were treated with 1 mL of chloroform: acetone (8:2, v/v) and vigorously vortexed. Then the tube was centrifuged at 20,000*g* for 10 min at 4°C. The protein pellet was dissolved in 2 × SDS sample buffer for further analysis. The supernatant was transferred to a new tube and evaporated under a stream of nitrogen. The resulting lipids were dissolved in chloroform for TLC analysis.

Collected bacteria were resuspended with 400 μL PBS. Then 400 μL methanol and 800 μL chloroform were added. The sample was vigorously vortexed 3 times for 1 min, with 1-minute intervals in between vortexing steps. Then the sample was centrifuged at 20,000*g* for 10 min at 4°C and the lower phase was transferred into a new tube. The remaining sample was extracted again with 800 μL chloroform. The two lipid solutions were combined and evaporated under a stream of nitrogen. The resulting lipids were dissolved in chloroform for TLC analysis.

The lipids were loaded on TLC plates and developed using solvent of hexane-diethyl ether-acetic acid (70:30:1, v/v/v). Then the TLC plate was stained with iodine vapor.

**Silver staining**

The gel was fixed for 30 min and transferred into sensitization solution for another 30 min. Following this step, the gel was washed four times for 5 min each, then incubated with silver nitrate solution for 20 min. The gel was rinsed briefly with double distilled water followed by incubation in 2.5% (w/v) anhydrous sodium carbonate solution until protein bands were conspicuous. The reaction was stopped with disodium ethylenediaminetetraacetic acid solution.

**Mass spectrometry study**

Proteins extracted from isolated LDs were dissolved in 8 M urea. For the protein bands indicated in Figure 3 and Figure S5, the bands were excised from gel, cut into small plugs, and washed twice with water. The gel pieces were destained with 40% acetonitrile/50 mM NH_4_HCO_3_, dehydrated with 100% acetonitrile and dried for 5 min using a Speedvac.

After reduction with dithiothreitol and alkylation with iodoacetamide, the samples were digested with trypsin at 37°C overnight. After quenching the reaction with formic acid, the peptides were desalted, followed by vacuum centrifugation. The resulting peptide mixtures were then analyzed by LC/MS/MS.

All analyses were performed on a nanoLC-LTQ-Orbitrap XL mass spectrometer (Thermo, San Jose, CA). For nanoLC, the Easy n-LC 1200 system was equipped with a 30 mm ReproSil-Pur C18-AQ (Dr. Maisch GmbH, Ammerbuch, Germany) trapping column (packed in-house, i.d.150 μm; resin, 5 μm) and a 150 mm ReproSil-Pur C18-AQ (Dr. Maisch GmbH, Ammerbuch, Germany) analytical column (packed in-house, i.d. 75 μm; resin, 3 μm). Solvents used were 0.5% formic acid water solution (buffer A) and 0.5 % formic acid acetonitrile solution (buffer B). Elution was achieved with a gradient of 5–10% B over 3 min, 10–28% B over 70 min, 28–40% B over 8 min, 40–100% B over 2 min, and 100% B for 7 min, at a flow rate of 300 nL/min.

Eluting peptide cations were converted to gas-phase ions by a Nanospray Flex ion source at 2.1 kV. The heated capillary temperature was 225°C. The mass spectrometer was operated in a data-dependent mode to switch automatically between MS and MS/MS. Survey full scan MS spectra were acquired from *m/z* 300 to *m/z* 1800, and the 10 most intense ions with charge state above 2 and above an intensity threshold of 500 were fragmented in the linear ion trap using a normalized collision energy of 35. For the Orbitrap, the AGC target value was set at 1e6 and a maximum fill time for full MS was set at 500 ms. Fragment ion spectra were acquired in the LTQ with an AGC target value of 3e4 and a maximum fill time of 150 ms. Dynamic exclusion for selected precursor ions was set at 90 s. The lock mass option was enabled for the 445.120025 ion.

The raw data were processed using Proteome Discoverer (version 1.4.0.288, Thermo Fischer Scientific). MS2 spectra were searched with SEQUEST engine against the *Rhodococcus erythropolis* PR4 and GCF_002155915.1_ASM215591v1 databases, for *Rhodococcus erythropolis* PR4 and *Streptomyces thermovulgaris*, respectively, and contaminant protein database. Database searches were performed with the following parameters: precursor mass tolerance 20 ppm; MS/MS mass tolerance 0.6 Da; two missed cleavages for tryptic peptides; methionine oxidation as a variable modification; cysteine carbamidomethylation as a fixed modification. The results were ﬁltered for a 1% false discovery rate (FDR) at the PSM level utilizing the percolator-based algorithm. Peptide identifications were grouped into proteins according to the law of parsimony.

The raw data of the main bands were subjected to proteomic analysis, and the major proteins were screened based on the Molecular Weight, PSMs and Unique Peptides. The data from the biological replicates of shotgun proteomes were screened for proteins with two unique peptides or above. All proteins identified were screened. Also, the identified proteins from the replicate analyses were combined and the NCBI database was used for functional categorization of the LD-associated proteins.


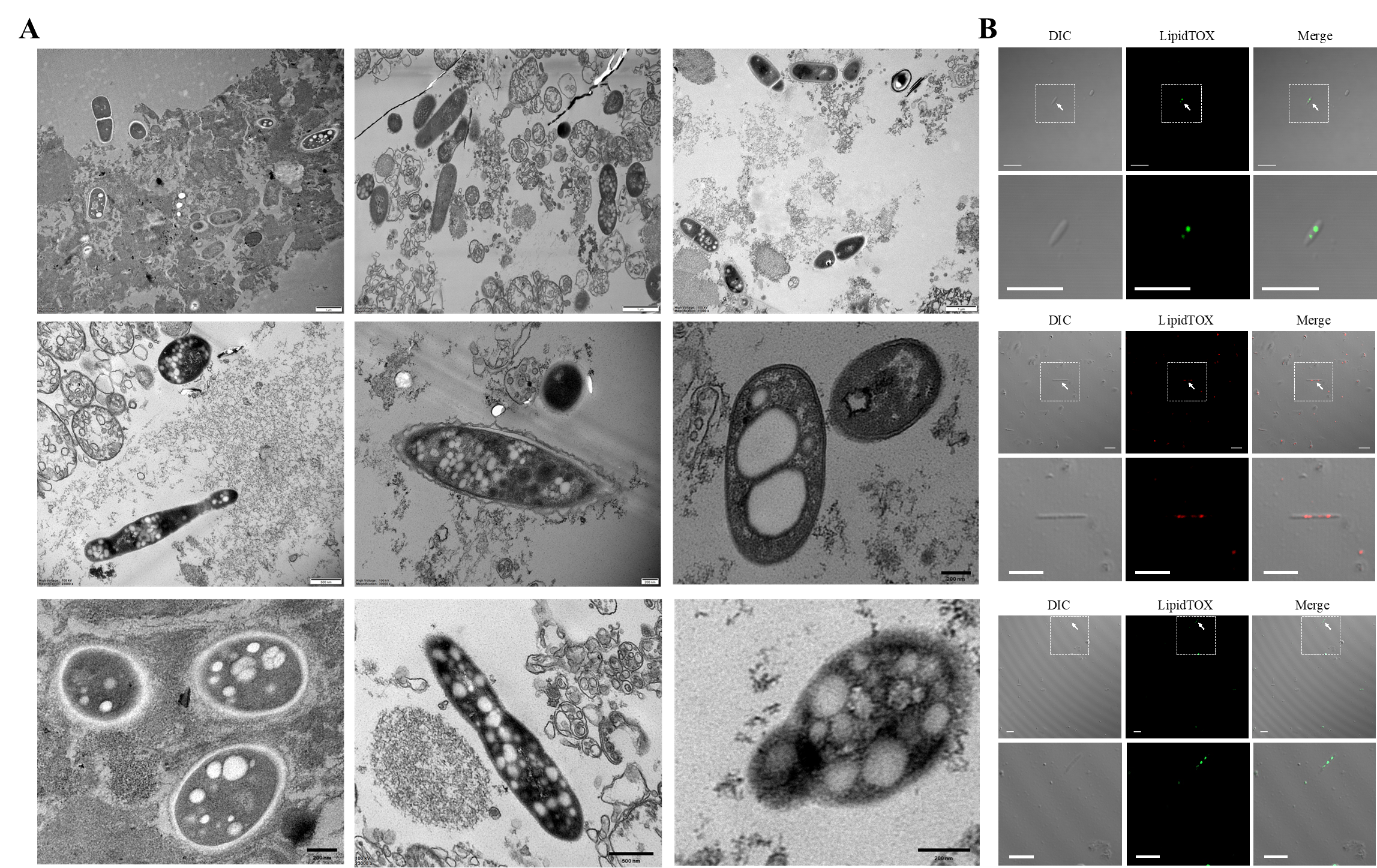


**Figure S1 Lipid Droplets in Mouse Small Intestinal Bacteria**

**A.** Ultra-thin structure analysis of fresh bacteria from mouse small intestine by TEM. **B.** The freshly-isolated bacteria from mouse small intestine were stained with LipidTOX Green or Red, and imaged by confocal microscope. Scale bar, 5 μm. Related to Figures 1Ca, 1Ce.


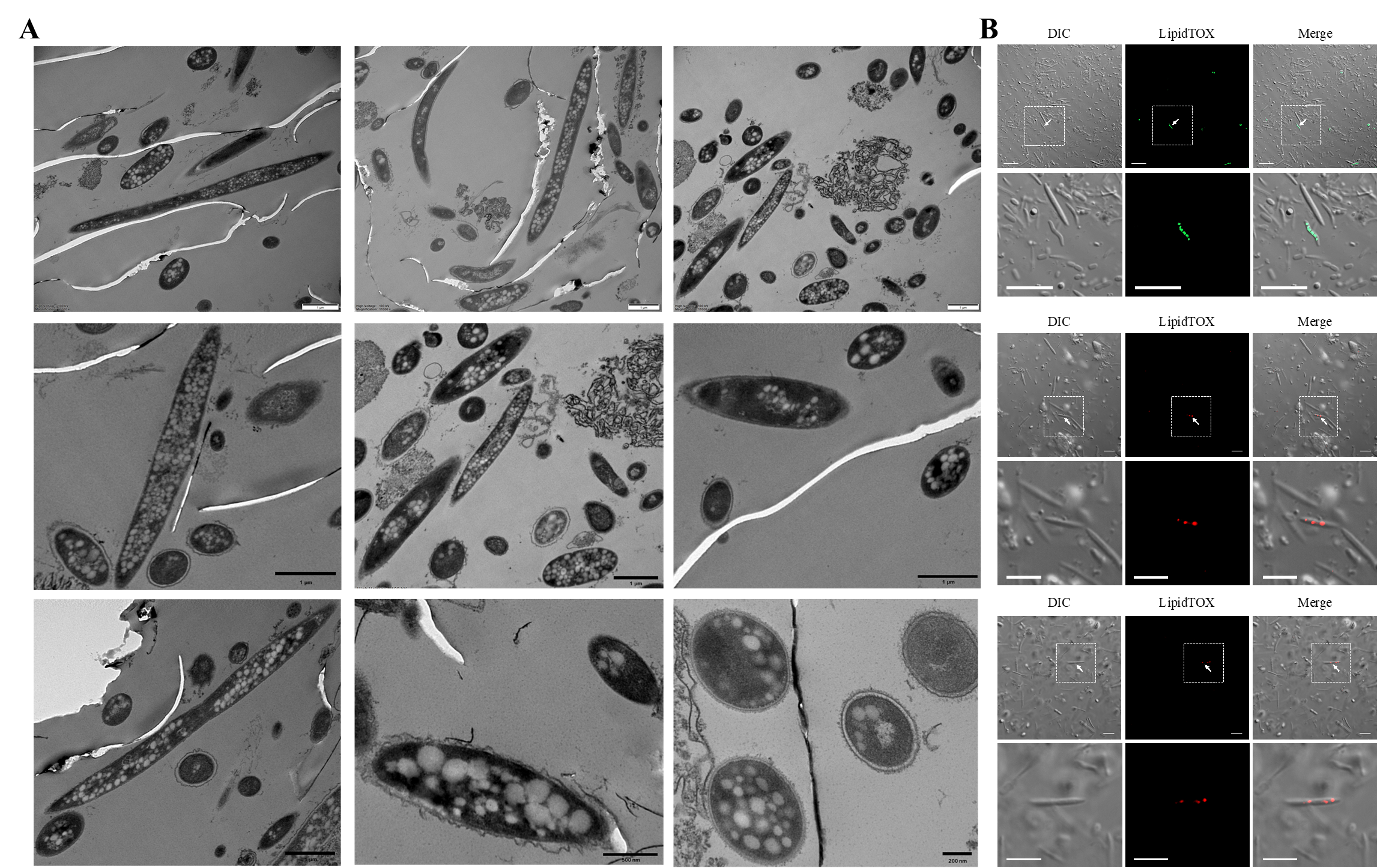


**Figure S2 Lipid Droplets in Mouse Large Intestinal Bacteria**

**A.** Ultra-thin structure analysis of fresh bacteria from mouse large intestine by TEM. **B.** The freshly-isolated bacteria from mouse large intestine were stained with LipidTOX Green or Red, and imaged by confocal microscope. Scale bar, 5 μm. Related to Figures 1Cb, 1Cf.


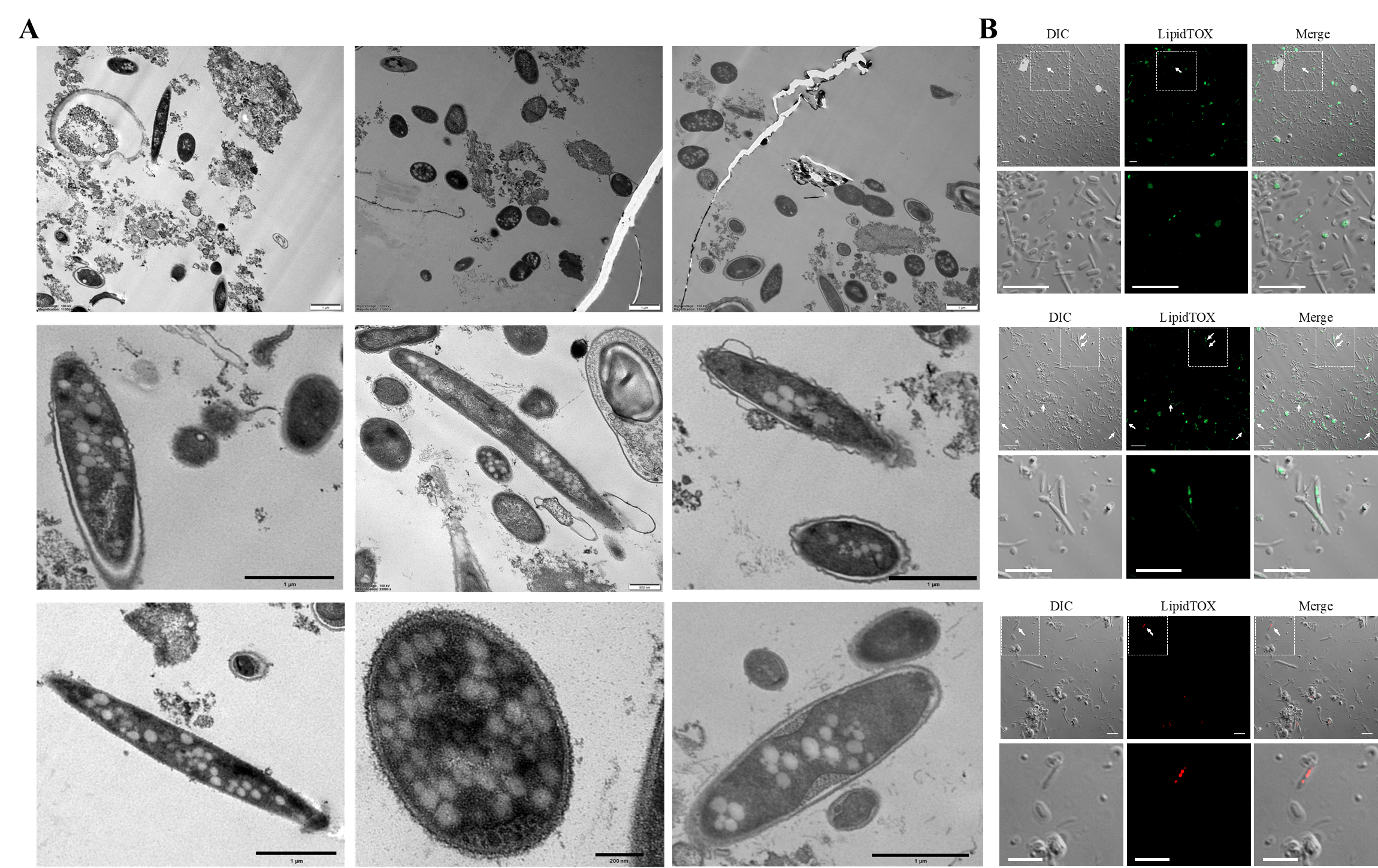


**Figure S3 Lipid Droplets in Mouse Fecal Bacteria**

**A.** Ultra-thin structure analysis of fresh bacteria from mouse feces by TEM. **B.** The freshly-isolated bacteria from mouse feces were stained with LipidTOX Green or Red, and imaged by confocal microscope. Scale bar, 5 μm. Related to Figures 1Cc, 1Cg.


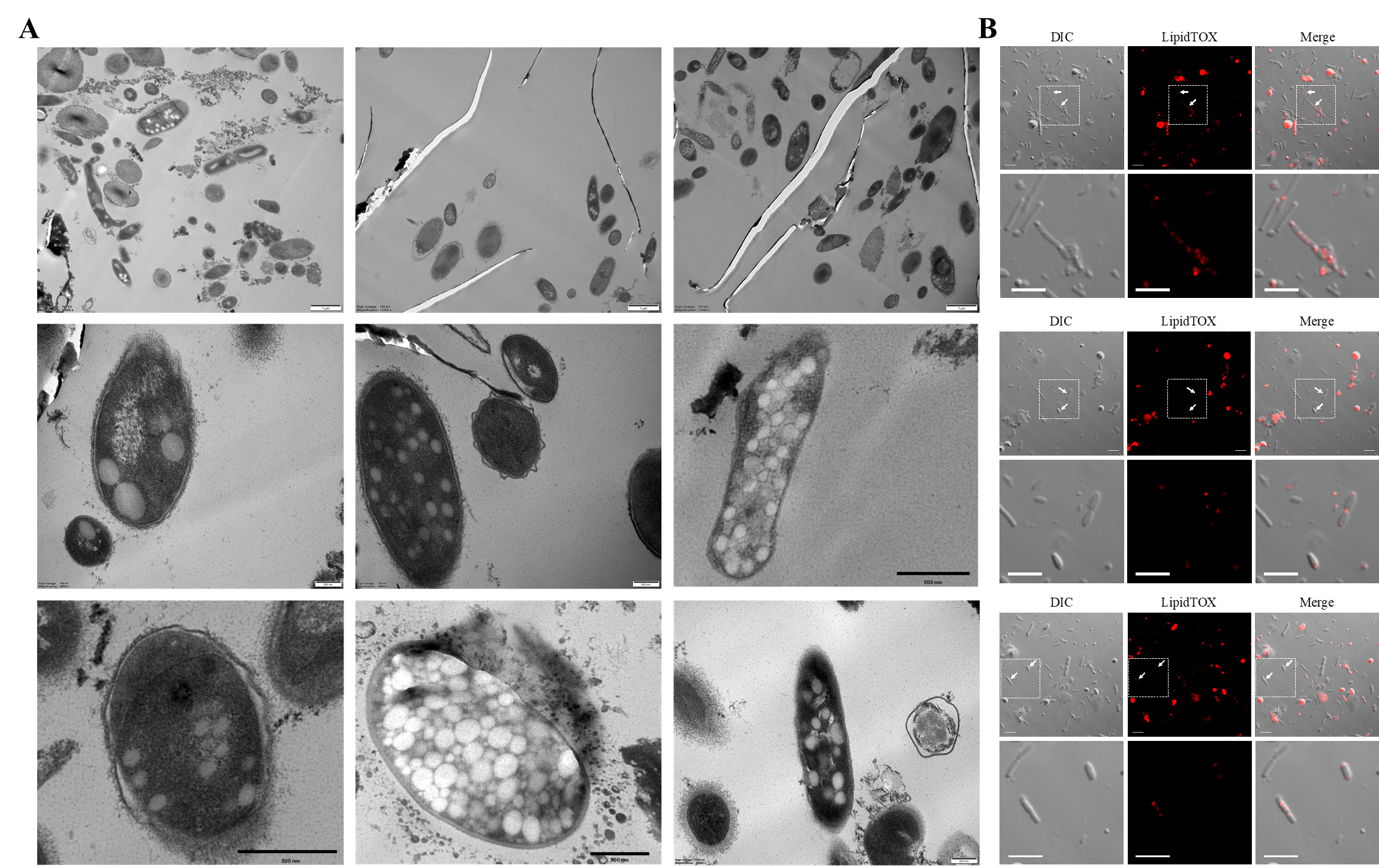


**Figure S4 Lipid Droplets in Human Fecal Bacteria**

**A.** Ultra-thin structure analysis of fresh bacteria from human feces by TEM. **B.** The freshly-isolated bacteria from human feces were stained with LipidTOX Red, and imaged by confocal microscope. Scale bar, 5 μm. Related to Figures 1Cd, 1Ch.


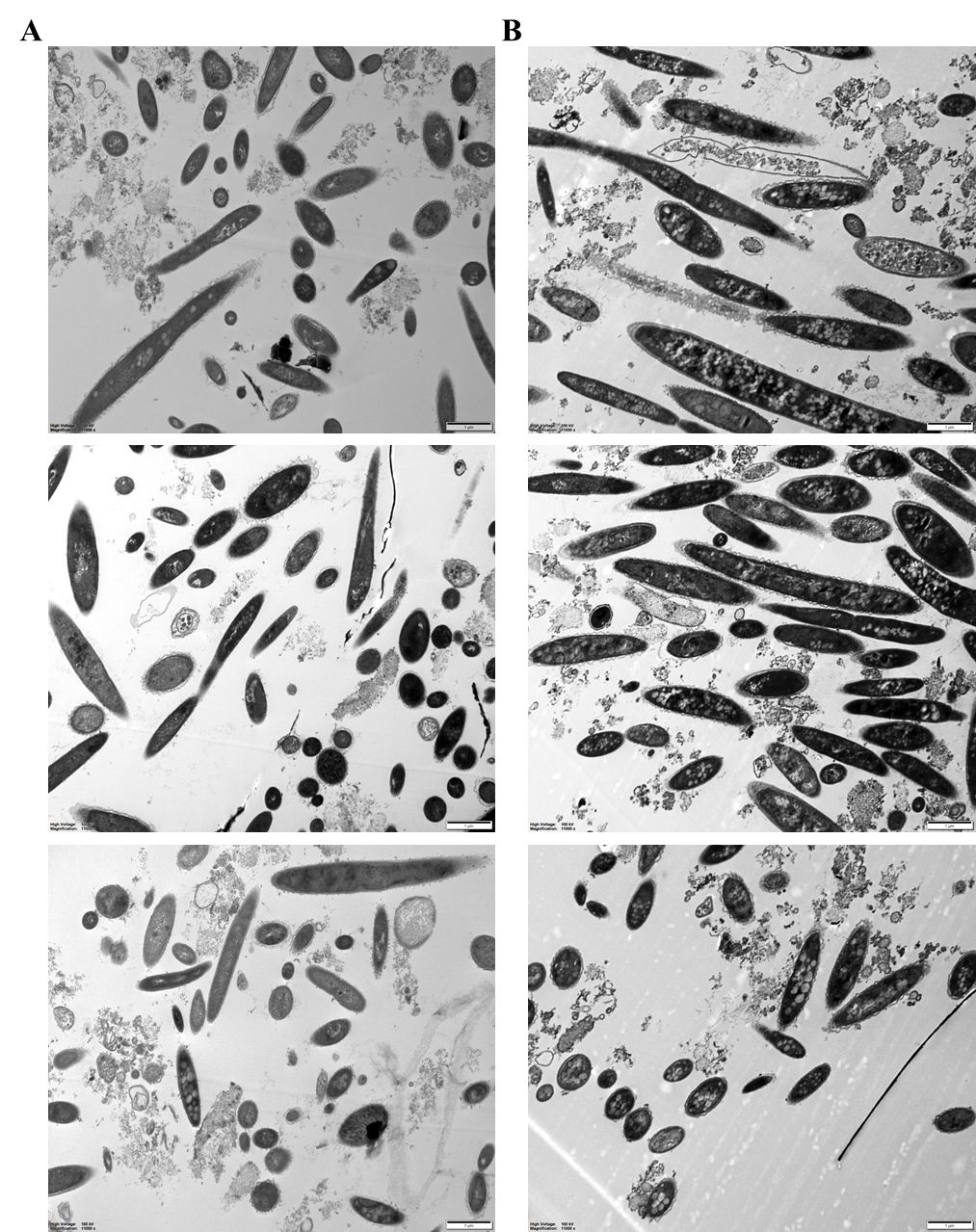


**Figure S5 Enrichment of LD-Containing Gut Bacteria**

After isolation of gut bacteria from mouse large intestine, the bacteria were separated further through Percoll density gradient centrifugation to enrich the subpopulation of gut bacteria containing LDs. The representative images of gut bacteria from mouse large intestine before (**A**) and after enrichment (**B**) are presented. Scale bar, 1 μm. Related to Figure 1D.


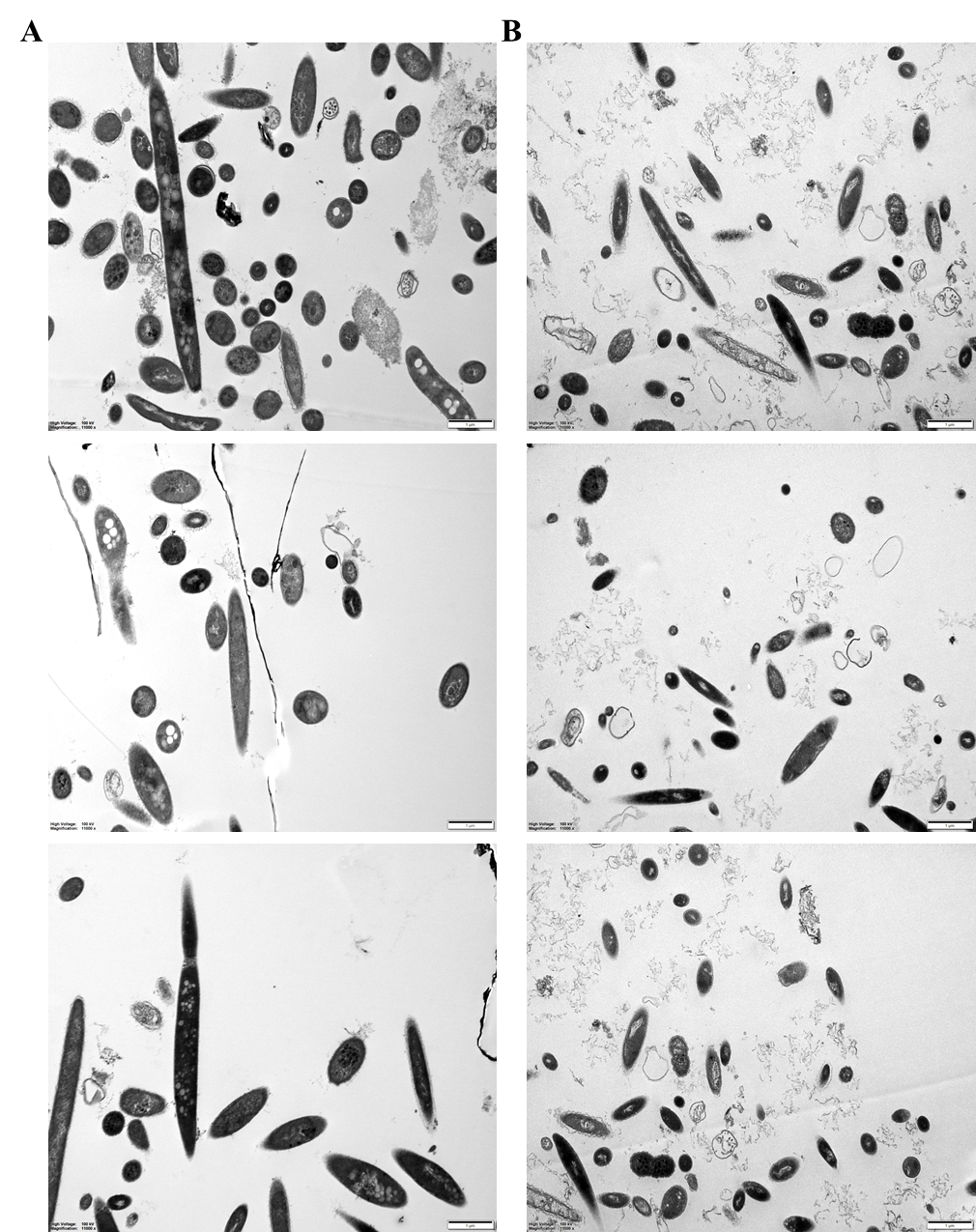


**Figure S6 Lipid Droplets in Gut Bacteria from Mice Fed Chow Diet and High-Fat Diet**

The representative images of isolated bacteria from mice fed chow diet (**A**) and HFD (**B**) are presented. Scale bar, 1 μm. Related to Figure 1E.


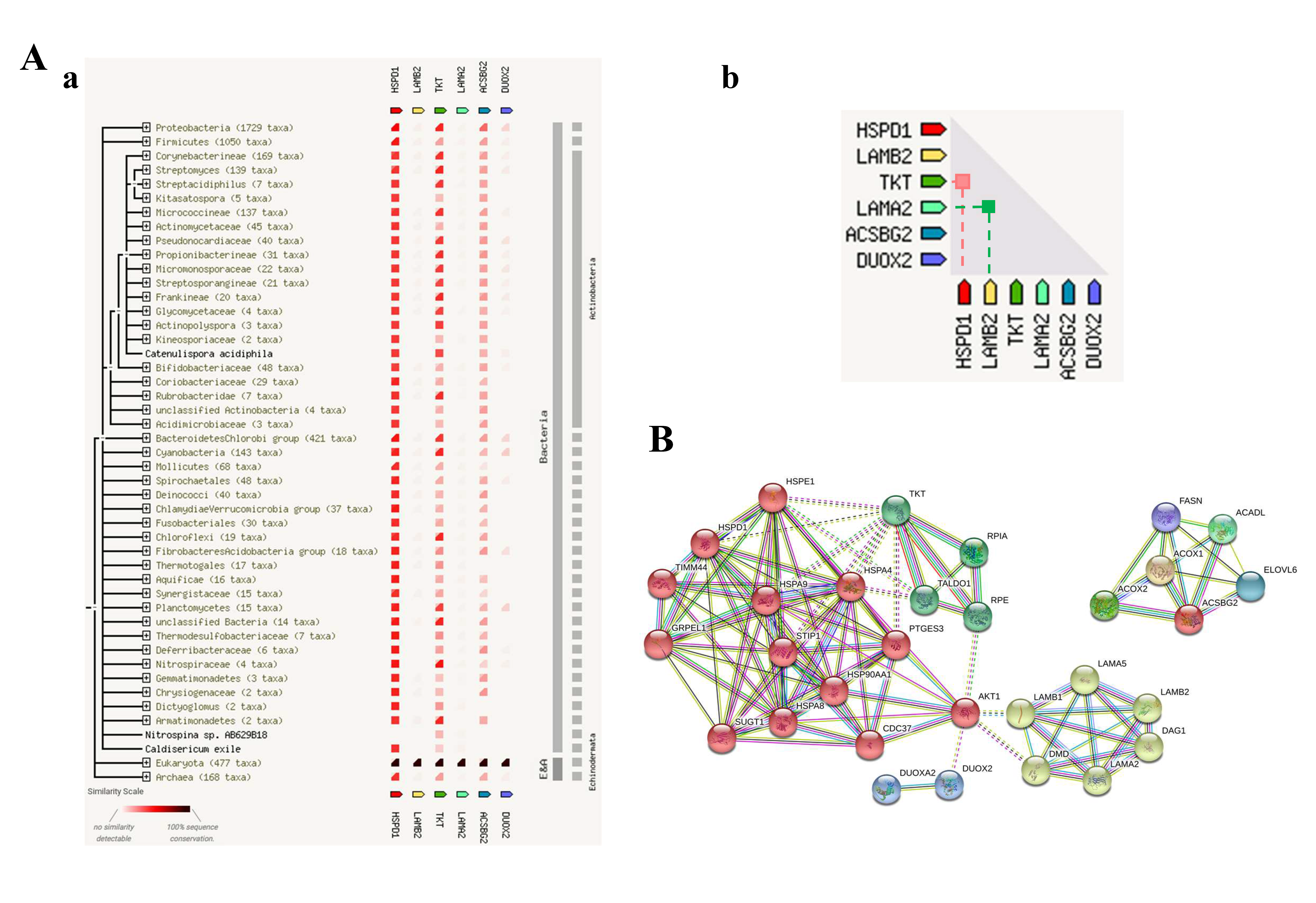


**Figure S7 Taxonomic Dissection and Functional Informatics Analysis of Several LD Abundant Proteins of Gut Bacteria**

Several abundant proteins were selected from proteomics results of gut bacterial LD and informatics analysis was performed. **A.** **a** Gene concurrence analysis was performed across different genomes from multiple domains of life including Bacteria, Archaea and Eukarya. The STRING database was used. For Eukarya, the human genome was taken as a reference. Dark red bars represent high gene distribution while light red represents low gene distribution. **b** Gene co-expression profiles among the selected genes were analyzed. Human was set as reference organism. **B.** The pathway-based protein network was analyzed for the six selected proteins. k-means clustering utilizing standard parameters was adopted.

**References**

1. Ott SJ, Waetzig GH, Rehman A, Moltzau-Anderson J, Bharti R, Grasis JA, et al. Efficacy of Sterile Fecal Filtrate Transfer for Treating Patients With Clostridium difficile Infection. Gastroenterology 2017; 152:799-811 e7.

2. Ding Y, Yang L, Zhang S, Wang Y, Du Y, Pu J, et al. Identification of the major functional proteins of prokaryotic lipid droplets. J Lipid Res 2012; 53:399-411.
